# Regional surveys of macrobenthic shelf invertebrate communities in Onslow Bay, North Carolina, U.S.A.

**DOI:** 10.1038/sdata.2018.54

**Published:** 2018-04-17

**Authors:** Carrie L. Tyler, Michał Kowalewski

**Affiliations:** 1 Miami University, Geology and Environmental Earth Science, 250 S. Patterson Av., Oxford, Ohio 45056, USA; 2 Florida Museum of Natural History, University of Florida, Museum Road, PO Box 117800, Gainesville, Florida 32611-7800, USA

**Keywords:** Biodiversity, Community ecology, Marine biology

## Abstract

Despite its importance for quantifying ecosystem responses to environmental and anthropogenic drivers, our understanding of spatial heterogeneity in marine communities remains inadequate. Studies in coastal marine benthic habitats are sparse, and predominantly target single higher taxonomic groups. Here we describe macrobenthic marine invertebrate community surveys from 52 localities in Onslow Bay (Beaufort, North Carolina, U.S.A.), over an extensive geographic area (~200 km^2^). The data consist of 11,467 individuals, 175 species, and 7 phyla. The data include species abundance data for each sample at all localities, and corresponding species lists and locality information. The metadata describe the sampling protocols and localities. The data provided here will facilitate examination of assemblage heterogeneity with regards to spatial and temporal patterns, and depth gradient analyses.

## Background & Summary

Mounting concerns about the ecological and societal implications of anthropogenic changes underscore the importance of identifying processes influencing biodiversity on multiple scales^1^ and understanding how and why community composition varies within and across habitats. Despite the need to understand the relationship between local and regional processes, ecosystem studies in coastal marine benthic habitats are sparse^2^ (but see^3–7^), typically concentrating on the deep-sea benthos and on single higher taxonomic groups, e.g., bivalves^8^, gastropods^9^, polychaetes^10–12^, bryozoans^4^, echinoderms^3,5^, decapods^13^, ascidians^14^, fish^15^, copepods^16^, isopods^17,18^, amphipods^19^, cumaceans^20,21^, and ciliated protozoa^22^. The macrobenthos is a key component of shallow marine ecosystems^23,24^, and biodiversity surveys are needed to examine spatial and temporal changes in community structure. Those changes, which may involve homogenization, local shifts in dominant fauna, or pollution gradients^2,6,7,25,26^, may indicate diverse ecosystem responses to variable anthropogenic pressures, particularly in coastal habitats. Assessments of regional ecosystem structuring may inform us as to the ecological impact of natural environmental drivers^11,22,27,28^ and/or anthropogenic pressures^2,6,7,25,26^.

This paper describes biodiversity survey data from shallow benthic marine communities in Onslow Bay (North Carolina, U.S.A) collected via dredging in 2011–2013. Field surveys were conducted to obtain quantitative community data including multiple phyla over an extensive geographic area (~200 km^2^) suitable for a variety of biodiversity analyses, particularly, how and why community composition varies within and across habitats (i.e., beta [β] diversity^29^ and other spatially explicit approaches). Shallow benthic marine community data including multiple higher taxonomic groups are sparse, and we hope that the data presented here will be widely utilized.

The current dataset should prove useful in understanding processes that influence biodiversity on multiple scales, and in developing effective strategies for conservation and coastal resource management^3,30,31^. The dataset described herein was collected in conjunction with sympatric death assemblage data^32,33^, for a project aimed at examining higher taxonomic fidelity of death assemblages (i.e., the incipient fossil record). Quantitative analyses of paleontological fidelity, based on comparisons of living communities to sympatric death assemblages, is an important research direction in paleobiology. To augment previous research, which has focused on single higher taxa (primarily mollusks), a comparative fidelity analyses across multiple groups of marine macrobenthos was carried out recently^32,33^. Using coastal and shallow subtidal settings of the Outer Banks (North Carolina, U.S.A.), fidelity and relative fossilization potential of multiple paleontologically important marine macro-invertebrate groups were assessed. A three-year, multi-site sampling program was carried out for quantitative live-dead comparisons of multiple major higher taxonomic groups of the macrobenthos. Fidelity was evaluated within and across higher taxa, and the relative preservational potential of major fossil groups was quantified in a comparative manner.

## Methods

**Figure 1:**
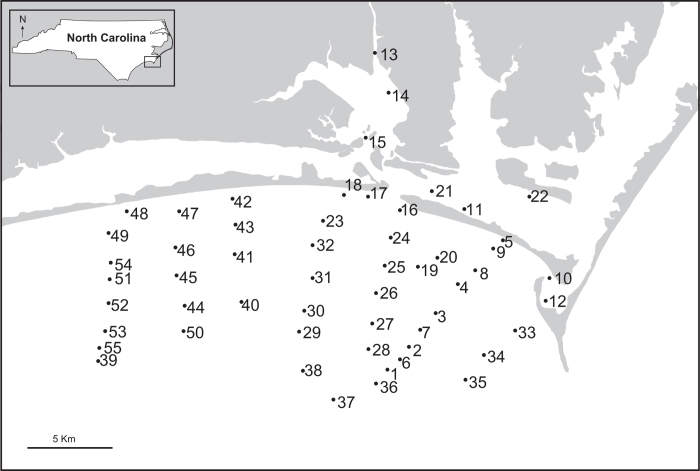
Study Area. Map of study area showing all 52 dredge localities where live surveys were conducted. Inset box in top left corner shows location of the field area within North Carolina. Numbers correspond to locality numbers in NC.Localities.xlsx (Data Citation 1) and NC.Abundance.xlsx (Data Citation 1).

Sampling targeted coastal estuarine and nearshore habitats near the city of Beaufort, North Carolina from November 2011 through March 2013. Marine benthic macro-invertebrate communities in the coastal and inner shelf habitats in Onslow Bay (Fig. 1) were extensively sampled, and samples were collected out to ~15 km offshore. The targeted field area was selected for its abundant and taxonomically diverse macrobenthos (both live and dead) that represents several major taxa important in the fossil record (multiple groups of benthic mollusks, regular and irregular echinoids, crustacean and chelicerate arthropods, corals, sponges, as well as annelids and brachiopods). Moreover, the study area offers easy logistic access to sampling localities including multiple habitats representing a gradient of depositional environments from coastal to shallow-shelf settings. Samples were collected between 76.545*°* N to 76.903*°* N, and 34.553*°* W and 34.800*°* W, an area where barrier islands and sandbars protect the coast of North Carolina from the open ocean (Fig. 1), and estuaries are somewhat sheltered from swells and storms. Water depth is relatively shallow on the shelf, and increases gradually to ~70 m with increasing distance from shore to the shelf break (~120 km off the coast), which marks a sudden dramatic increase in depth. Nearshore sediments are fine sands, and at depths>10 m sediments are variable, including medium to coarse sands (0.25–2 mm), and gravel^34^. Sediments in the back sounds are typically fine to medium sand (0.50–0.063 mm) near beaches, or silt (0.063–0.004 mm) and clay (<0.004 mm) in quiet or deep water^35^.

The coast consists of a broad, shallow, high-energy shelf environment, where beach profiles are bedrock controlled and the modern sediment layer is relatively thin (0–1.5 m). Strata slope to the east and southeast and the region is overlain by Holocene sand that thinks seaward. Pliocene limestone underlies the Quaternary strata at Shackleford banks, at ~23 m below the sediment surface. The shallow Quaternary stratigraphy of Shackleford and Bogue Banks is dominated by a regressive succession consisting of inlet fill deposits overlying Pleistocene and Neogene shoreface sediments. Erosion during the recent transgression has truncated the Pleistocene strata, leaving a thin layer of Holocene coastal sediment^36^. These thin beach sediments are dominated by medium-fine grained sand, consisting of shell hash, granule size quartz grains, and silt. The shoreface consists of a microtidal environment with a mean tidal range of 0.97 m^37^ and semidiurnal tides. Wave energy is predominantly from the southeast during summer and the northeast during winter. Storm activity is concentrated in September – February. Average open marine salinity in the region is 36 ppt, with the warm Gulf Stream flowing from the south. Nearshore salinity averages 34 ppt and estuarine waters have variable salinities dependent on precipitation. Inner shelf water temperature varies seasonally (> 28 °C in summer, 12–14 °C in winter).

**Figure 2:**
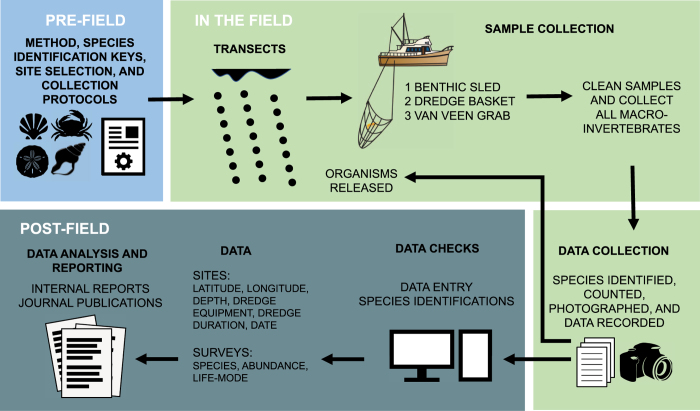
Phases of data collection. Workflow of study design, sampling, data collection, data processing, and reporting compartmentalized according to pre-field, field, and post-field activities for data published here.

Sampling was designed to capture the spatial variation of multiple higher taxonomic groups of invertebrates in an area containing several depositional environments from coastal to shallow-shelf settings (Fig. 2). 43 Localities were therefore selected to form seven open marine onshore-offshore transects, with localities relatively equidistant along the transect (Fig. 1). Due to the curvature of the coastline, not all transects are parallel (Fig. 1), and 9 additional localities were randomly selected to capture estuarine habitats. Three types of dredging equipment were employed at each locality during each field season to ensure an adequate representation of multiple types of benthic organisms, including shallow infaunal species. Spatial distribution of benthic organisms between sampling units (localities) was assumed to be patchy. Thus, localities were spaced at ~2 km distance from each other to ensure spatially adequate estimates of species richness and dominance patterns^38–40^. Dredging was conducted at 52 localities over four field seasons: June 2011, November 2011, May of 2012, and April 2013 (Table 1 (available online only)), resulting in a total of 220 benthic invertebrate dredge samples collected at 52 localities representing a variety of habitats, depths, and distances from shore (Fig. 1). A total of 36 localities were sampled during a single field season, while 13 localities were sampled twice (i.e., in two different field seasons), and 3 localities were sampled three times (i.e., in three different field seasons). Locality and sampling data are provided in NC.Locality.xlsx (Data Citation 1).

During a given field season, at each locality a minimum of three samples were collected, one sample for each of the three types of equipment deployed: a benthic sled, a dredge basket, and a van Veen grab. The sled trawling duration at each locality was 5 min, and the basket trawling duration was 10 min. Equipment was deployed while the vessel was stationary (starting point). Once the equipment was on the seafloor, and the vessel began to move dragging the equipment perpendicular to shore, trawl time began, and trawling ceased once the allotted time had passed, either 5 or 10 min depending on the equipment deployed (ending point). For each sample, maximum depth from the surface was recorded using the onboard depth sounder (±0.3 m). The benthic sled was lined with 1 mm wire mesh to ensure representative sampling of smaller species and juveniles, and van Veen samples were wet sieved (1 mm mesh).

Samples were examined to extract all live invertebrates that were identifiable, with the exception of encrusting species (such as bryozoans and some sponges). All specimens were counted and identified to the lowest taxonomic level (typically species). The resulting live surveys consists of 220 samples with 175 species from 7 Phyla (Annelida, Arthropoda, Brachiopoda, Cnidaria, Echinodermata, Mollusca, and Porifera). Species names associated with numeric codes are listed in NC.Species.List.xlsx (Data Citation 1). All data processing was conducted in R 3.4.3 using base functions provided^41^. The software is available from https://www.r-project.org/.

## Data Records

These data are distributed under the Attribution 4.0 International (CC BY 4.0; http://creativecommons.org/licenses/by/4.0/). Users are required to cite this data paper in any resulting publication or report, however, users are free to share and adapt/analyze the data for any purpose, even commercially, providing there is attribution to the original data and any changes are detailed. The data are downloadable as three.xlsx files from the Dryad online repository. The files contain (1) details of localities and samples can be found in NC.Localities.xlsx (Data Citation 1), (2) species lists and numeric species codes for each of the 175 species are provided in NC.Species.List.xlsx (Data Citation 1), and (3) the species abundance by sample in NC.Abundance.xlsx (Data Citation 1) which consists of 4 columns and 1870 rows (Table 1 (available online only)). Each species was assigned a reference number, listed in NC.Species.List.xlsx (Data Citation 1). Similarly, each locality was assigned a number, listed in NC.Localities.xlsx (Data Citation 1) and marked on the study area map (Fig. 1), corresponding to locality numbers in the species abundance data.

At each locality, the following is provided: (i) date of sample collection, (ii) unique locality number, (iii) sample number, (iv) dredge equipment used, (v) maximum depth (m), (vi) minimum depth (m), (vii) starting latitude (decimal degrees), (viii) starting longitude (decimal degrees), (ix) ending latitude (decimal degrees), (x) ending longitude (decimal degrees), and (xi) duration (minutes). Taxa that live within the sediment were classified in the species list as ‘infuanal’. Epibenthic taxa that live on the surface of the substratum were classified as ‘epifuanal’. Taxa that live partially within the sediment, but are also partially exposed above the sediment-water interface were classified as ‘semi-infaunal’. Taxa that were unique, but could not be identified to genus and/or species are denoted by a period (i.e., missing data) under ‘Genus’ and/or ‘Species’. Samples collected using a Van Veen Grab consist of a point sample with a single depth (recorded as maximum depth) and GPS location (recorded as starting latitude and longitude). These samples therefore do not have a minimum depth, end latitude, end longitude, or duration, all of which are missing and denoted by ‘NA’.

The data format for each file is as follows:

NC.Localities.xlsx (Data Citation 1)

Column 1: “Date” - Sample Collection Dates

Column 2: “Locality” - Locality Number (corresponds to “Locality” in NC.Abundance.xlsx)

Column 3: “Sample” - Sample Number (corresponds to “Sample” in NC. Abundance.xlsx)

Column 4: “Equipment” - Type of Dredge Equipment Deployed

Column 5: “Max.Depth” - Maximum Dredge Depth (meters)

Column 6: “Min.Depth” - Minimum Dredge Depth (meters)

Column 7: “StartLat” - Starting Latitudinal GPS Coordinates (decimal degrees)

Column 8: “StartLong” - Starting Longitudinal GPS Coordinates (decimal degrees)

Column 9: “EndLat” - Ending Latitudinal GPS Coordinates (decimal degrees)

Column 10: “EndLong” - Ending Longitudinal GPS Coordinates (decimal degrees)

Column 11: “Duration” - Dredge Duration (minutes)

NC.Species.List.xlsx (Data Citation 1)

Column 1: “Species_ID” - Numeric Species Code (corresponds to “Species_ID” in NC. Abundance.xlsx)

Column 2: “Phylum”

Column 3: “Class”

Column 4: “Genus”

Column 5: “Species”

Column 6: “Life Mode”

NC.Abundance.xlsx (Data Citation 1)

Column 1: “Locality” - Locality Number (corresponds to “Locality” in NC.Localities.xlsx)

Column 2: “Sample” - Sample Number (corresponds to “Sample” in NC. Localities.xlsx)

Column 3: “Species_ID” - Numeric Species Code (corresponds to “Species_ID” in NC. Species.List.xlsx)

Column 4: “Abundance” - Abundance of each species within a sample

## Technical Validation

Sample collection methods were standardized for all localities. At each locality, the same three types of dredge equipment were deployed and trawl durations were timed and held constant (when appropriate). Each locality that was sampled during a single field season, therefore, consists of a minimum of three samples (one sample with each equipment type). However, live specimens were not found in all samples (thus, the number of samples at some localities are not multiples of three as a result of samples with no organisms). To standardize sampling effort across localities, a standard trawl duration was used for each type of bottom trawling equipment. Trawling ceased once the allotted time had passed, either 5 or 10 min depending on the equipment deployed. The sled trawling duration at each locality was 5 min, and the basket trawling duration was 10 min. Only 8 samples deviated from these times with trawl durations provided for each locality in NC.Locality.xlsx (Data Citation 1). All species identifications were conducted by a single individual (PI Tyler), using several keys^42–44^. All nomenclature was verified in the World Register of Marine Species^45^. Voucher specimens are housed at the Florida Museum of Natural History in the Invertebrate Zoology research collections. Overall, assemblages included adequate representation of infaunal organisms (27% of species were infaunal). Although previous analyses conclude that seasonal variability in community composition is negligible in this region^46^, 31% of localities were sampled repeatedly in different seasons to reduce potential seasonal effects and improve locality-level estimates of richness, diversity, and relative species abundances.

Sampling was conducted in accordance with regulations and guidelines outlined by the Duke University Marine Lab (DUML), and fell under the DUML invertebrate collections permits (DUML Scientific or Education Permit 707075 for 2011, 2012, and 2013). With the exception of voucher specimens, all individuals were released *in situ* after counting and identification. No protected species were identified in the sampled material.

## Additional information

**How to cite this article**: Tyler, C. L. & Kowalewski, M. Regional surveys of macrobenthic shelf invertebrate communities in Onslow Bay, North Carolina, U.S.A. *Sci. Data* 5:180054 doi: 10.1038/sdata.2018.54 (2018).

**Publisher’s note**: Springer Nature remains neutral with regard to jurisdictional claims in published maps and institutional affiliations.

## Supplementary Material



## Figures and Tables

**Table 1:** Metadata Records

**Locality**	**Samples**	**Field Seasons**	**June 2011**	**Nov. 2011**	**May 2012**	**April 2013**	**Depth (m)**	**Latitude (decimal degrees)**	**Longitude (decimal degrees)**
1	3	1	0	3	0	0	18.9	−76.67457	34.57305
2	5	2	0	4	1	0	18.7	−76.66083	34.58407
3	8	2	0	5	3	0	18.2	−76.63953	34.60843
4	7	2	0	4	3	0	16.6	−76.62340	34.62818
5	4	1	0	4	0	0	11.6	−76.58260	34.65860
6	4	1	0	4	0	0	18.4	−76.66190	34.58105
7	4	1	0	4	0	0	18.4	−76.64855	34.59813
8	7	2	0	3	4	0	15.2	−76.60395	34.63938
9	5	2	0	2	3	0	7.9	−76.57627	34.65788
10	3	1	0	3	0	0	8.2	−76.54587	34.63763
11	7	2	0	5	2	0	9.3	−76.61390	34.67830
12	4	2	0	1	3	0	8.7	−76.54757	34.62415
13	5	2	0	3	2	0	5.3	−76.68693	34.80010
14	8	3	1	4	3	0	5.8	−76.67603	34.76252
15	8	3	2	3	3	0	5.5	−76.69370	34.72408
16	11	2	0	5	6	0	13.6	−76.66548	34.68128
17	6	1	0	6	0	0	6.6	−76.68642	34.68845
18	8	2	0	5	3	0	7.8	−76.70923	34.69100
19	6	2	0	4	2	0	17	−76.64442	34.67498
20	4	1	0	4	0	0	15.2	−76.63438	34.65047
21	8	3	2	3	3	0	8.4	−76.63570	34.69213
22	7	2	0	4	3	0	5.1	−76.56282	34.68957
23	3	1	0	0	3	0	16	−76.72652	34.67292
24	3	1	0	0	3	0	15.2	−76.67270	34.66258
25	3	1	0	0	3	0	16.4	−76.67737	34.64325
26	3	1	0	0	3	0	17.1	−76.68113	34.62642
27	3	1	0	0	3	0	17.9	−76.68563	34.60527
28	3	1	0	0	3	0	18.4	−76.68885	34.59005
29	6	2	0	0	3	3	17.7	−76.74262	34.59920
30	3	1	0	0	3	0	17.4	−76.74145	34.61365
31	3	1	0	0	3	0	16.2	−76.73930	34.63697
32	3	1	0	0	3	0	17.1	−76.73627	34.65765
33	3	1	0	0	0	3	16.3	−76.57508	34.59567
34	3	1	0	0	0	3	17.9	−76.59653	34.58213
35	2	1	0	0	0	2	18	−76.61100	34.56682
36	2	1	0	0	0	2	18.9	−76.68190	34.56535
37	2	1	0	0	0	2	19	−76.71693	34.55383
38	3	1	0	0	0	3	18.4	−76.74387	34.57403
39	3	1	0	0	0	3	17.1	−76.74387	34.57403
40	3	1	0	0	0	3	16.9	−76.79652	34.62135
41	5	1	0	0	0	5	16.9	−76.80327	34.64753
42	4	1	0	0	0	4	13.3	−76.80017	34.68582
43	4	1	0	0	0	4	16.5	−76.80238	34.66863
44	5	1	0	0	0	5	17	−76.83778	34.61485
45	3	1	0	0	0	3	16.9	−76.84503	34.63537
46	3	1	0	0	0	3	16	−76.84560	34.65510
47	3	1	0	0	0	3	13.2	−76.84283	34.67935
48	3	1	0	0	0	3	11.5	−76.88408	34.67898
49	3	1	0	0	0	3	15.8	−76.90047	34.66692
50	3	1	0	0	0	3	17	−76.83987	34.59897
51	10	1	0	0	0	10	17.1	−76.90308	34.64302
52	3	1	0	0	0	3	17	−76.90005	34.61767
The number of samples (‘Samples’) collected at each locality in each field season (June 2011, November 2011, May 2012, and April 2013). Latitude and longitude are in decimal degrees.									
